# Towards Establishing Fiscal Legitimacy Through Settled Fiscal Principles in Global Health Financing

**DOI:** 10.1007/s10728-015-0305-z

**Published:** 2015-09-04

**Authors:** Attiya Waris, Laila Abdul Latif

**Affiliations:** Commercial Law Department, Law School, University of Nairobi, Nairobi, Kenya; Go4Health Project, University of Nairobi, Nairobi, Kenya

**Keywords:** Health financing, Tax law, Tax policy, Canons of taxation, Africa, International health financing

## Abstract

Scholarship on international health law is currently pushing the boundaries while taking stock of achievements made over the past few decades. However despite the forward thinking approach of scholars working in the field of global health one area remains a stumbling block in the path to achieving the right to health universally: the financing of heath. This paper uses the book Global Health Law by Larry Gostin to reflect and take stock of the fiscal support provided to the right to health from both a global and an African perspective. It then sets out the key fiscal challenges facing global and African health and proposes an innovative solution for consideration: use of the domestic principles of tax to design the global health financing system.

## Introduction

When a human being is unhealthy they cannot work. The French constitution of 1793, states that the human being is owed subsistence and by extension this includes ensuring care during ill health [9]. Despite this centuries-old principle, inadequate funds for health services continue to be a global problem for states, and Africa is no exception. The recent Ebola crisis also triggered debates on an international health emergency fund. In the wake of the 2009 global fiscal crisis states with social services and national health care systems, governments and ministries have begun and continue to scale back services: excluding individuals, reducing types of services and their quality and closing institutions. In Africa, there is periodic lack of medicines at clinics, staff strikes and privatization of health care through national health insurance schemes. This reduction in state financed health care services is coupled with increased taxation without reflected increase in health spending and encouraging insurance which translates into double payment by individuals for a reduced health service.

The International Covenant on Economic, Social and Cultural Rights (ICESCR) sets out the right to health and all African states have signed and ratified it. This paper will address health care from a rights based perspective. The political will of African leaders to put health at the forefront of development has been reiterated at the continental level not only through ratification of the ICESCR but also through actions such as the Abuja Declaration of 2001 on increasing government funding for health, the Addis Ababa Declaration of 2006 on community health in the African Region, the 2008 Ouagadougou Declaration on Primary Health Care and Health Systems in Africa [52] and the 2012 Tunis Declaration on value for money, sustainability and accountability in the health sector. In practice, joint ministry of health and finance discussions revealed consensus that universal health coverage (UHC) requires increased funding, and more efficient use of existing resources [25]. This is the first concern of the paper: the financing of global and regional health.

The lost state revenue discovered through whistle-blowers is in the billions of US dollars from Africa alone [13]. International scandals like Luxleaks [21], Swissleaks [22] and the Google [3] tax avoidance scheme points that: one, there are resources available; two they are not being collected efficiently and effectively and three, there is injustice in the national, regional and global fiscal systems. Despite paying taxes, beyond their ability, the stress of taxes and finance is being placed on the unhealthy individual and his/her family and friends at a grass root level.

Tax compliant individuals and institutions must trust the state building its fiscal legitimacy. Fiscal legitimacy requires that a fiscal system must respond to principles of good governance (transparency, accountability and responsibility) as well as being an effective, efficient, fair and just system [47]. To achieve fiscal legitimacy, principles and canons of taxation are used in national budgets and fiscal systems. There is no similar guide for global financing generally and health specifically. The details of the financing of global and regional health, its guiding principles and structure for the collection and distribution of resources for the realisation of the right to health potentially through the canons of taxation, is therefore the second concern of this paper.

Gostin’s book discusses part of the first concern of this paper: the entire system of global health law and importance of global health with limited reference to Africa. He charts the path on financing health indirectly tracing the development of global health and health systems highlighting the difficulties [14, 35]. On the second concern of this paper, Gostin makes reference to financing health from the perspective of existing challenges with health aid [14, 35] and innovations like the health impact fund [14, 35]. However, he does not discuss fiscal legitimacy and financing global health. International co-operation and assistance is only now being gradually unpacked in order to understand what it could and should entail [26].

This paper will continue the discussion on financing health from where Gostin ends to consider whether domestic principles of taxation can guide international health financing while reflecting on the practice in Africa. This paper is divided into five parts: part one introduces the issue; two sets out fiscal legitimacy and the principles of taxation as a potential guide in global health systems. Part three sets out the current global and African practices in health financing; four recommends and five concludes.

## Conceptually Linking Tax to the Right to Health

Conventions, Declarations, resolutions and comments set out the minimum standards that states agree to be bound by. By participating in the international human rights framework, states agree to undertake that constitutions, laws, policies, and budgets reflect these legal obligations and policies are applied towards achieving these minimum standards [37]. However, financing remains within state control.

The World Health Organisation (WHO) states that the right to health [45] requires governments to generate conditions where everyone is as healthy as possible. This right is found in the Convention on the Rights of the Child, ICESCR, and the Convention on the Elimination of All Forms of Discrimination against Women. It encompasses, provision of medical care and underlying determinants of health [11].

At the global level, there is a difference between countries’ health financing needs and their current health spending [15]. This can be seen from the differences in the financing gap between high-income countries, middle-income countries and low-income countries. Low-income countries carry 90 % of the world’s health burden, yet only 12 % of global health spending occurs in these countries [15]. In addition, low-income countries account for 12 % of global GDP. There has been little commitment towards bridging the financing gap between the high income and low income countries. Conventional options at the global level reflect the domestic level financing options such as taxation, user fees and insurance, with limited innovation. A set of principles guiding global health financing is overdue.

This section delves into concepts to guide global health financing. Within health this includes: firstly, stating that resources are necessary in order to achieve rights; and secondly, unpacking international co-operation and assistance. Within the financing domain, there is a need to firstly, delineate fiscal legitimacy within the context of health; and secondly, discuss the principles and canons of taxation that could be used to fill in the gap in the global and regional health financing architecture.

### Rights Require Resources

In theory, when a state signs an international covenant like the ICESCR that contains the right to health, it takes on the duty to protect, promote and respect the right [10]. This duty has been set out in the commencing clause of the UN Charter [38], the Tehran Declaration [40], the Vienna Declaration [43] and most recently in 2004 by a General Assembly Resolution [2] through General Comment 31 where it stated at paragraph 5 that the obligations of the Covenant and article 2 are binding on every State Party as a whole and all its branches of government [44]. There was however, no further step taken to concretize its fiscal implications. All declarations avoided mention of finance or resources and instead used immediate or progressive realization. This resulted in states like those in Africa prioritizing rights that fall under immediate realization.

Reference to the issue of resources, especially domestic resources like tax revenue and its allocation was avoided on the grounds of the sovereignty of nations and to allow states to pursue independent domestic economic policies. General Assembly resolutions reaffirmed that states should not interfere in the domestic policies of other countries [39, 41]. It was not until the declaration on the Right to Development that there was finally crystallization of the principle that there was a need for resources and that ideals were no longer enough [42]. This declaration was spearheaded by developing countries including many African states as they recognized the lacuna.

From a fiscal perspective, the absence of a directive on the need for resources meant that ministries of finance ignored it in budgets. However rights that impose duties, responsibilities and obligations on a state by necessity require resources. International treaties could only become more than mere declarations if they would confer fiscal power on bodies whose decisions are legally binding. People, not living in a state having effective government policies supporting rights, would have no remedies and in reality have no legally, fiscally enforceable rights. In finance, legal rights exist in reality when and if they have budgetary support. If the state claims to grant the right to free health for example, this will only take place on the ground if there are adequate resources to build clinics near communities. This conceptual lacuna in the human rights principles that stopped short of stating resources were required has impeded national realization of the right to health as it relies solely on political will.

### Unpacking International Assistance and Co-operation

The ICESR went a step further than the ICCPR and stated at article 2(1):

Each state party to the present covenant undertakes to take steps, individually and through international co-operation especially economic and technical, to the maximum of its available resources, with a view to achieving progressively the full realization of the rights recognized in the present Covenant by all appropriate means, including particularly the adoption of legislative measures [20].

Analysis of the specific term in the article: international assistance and co-operation leads to terms like ‘limitation to resources’ or resource ‘constraints’. These terms are used repeatedly while espousing ‘progressive realization’ of human rights ‘obligations’ [19]. These terms are used by states to delimit and exclude themselves from domestic level human rights obligations, limit and avoid international obligations for assistance and co-operation. For example ODA states have internationally committed to giving 0.7 % of GDP per state, in reality not all states contribute ODA and only four states periodically reached that figure [32].

The WHO’s objective is the attainment by all peoples of the highest possible level of health and it is mandated with directing and coordinating global health work [54]. This includes partial responsibility for correcting the financial mismatch in health between states. At the global level, realization of the right to health as a membership based organisation would mandate it to make spending decisions based on a majority vote and form a large part of international co-operation and assistance. With the discussions on large-scale tax evasion and avoidance, suggestions of new taxes like the transaction tax, [14, 35] and funds like Pogge’s health impact fund [14, 35] are also being tabled. International co-operation and assistance could assist states in collecting untapped resources and newly created global taxes.

### Fiscal Legitimacy in Achieving Health

Increasing resources and spending is not enough to fully realize the right to health. Application of fiscal principles in the implementation practices and policies within international co-operation and assistance is required to ensure fiscal legitimacy. Legitimacy arises out of the confidence of the ruled and fiscal legitimacy arises out of the confidence in the fiscal behaviour of the governing institution of the fiscal resources placed in its trust [50]. Using international co-operation, at the level of states and a governing body like the UN or the WHO that act on states’ behalf there must be similar level of trust.

Firstly, citizens, both individually and as a society (and a state as a collective representative) perceive taxation or other payments as one, a necessary burden to the state (and by extension a governing interstate agency or institution) and two, as having no commensurate benefits or guarantees [6]. The concept of ‘necessary burden’ has resulted in revenue collection disparities in countries, as the relatively large size of the economy is not reflected in the proportionately small amount of tax revenue collected. There are numerous reasons for this, including but not limited to the societal perception of tax as a burden leading to both avoidance and evasion of tax. The effects of both domestic and global policies, which allow inputs of diverse actors as well as poor governance practices, fuel this perception. Domestically all who owe taxes must pay in full and on time or pay penalties and internationally necessary burden requires that states forming the international institution must fulfill their obligations.

Secondly, the perception of poor governance fuels perceptions of the lack of accountability, transparency and responsibility of the state (or international institution) in collection and use of resources by citizens, taxpayers and society. This is commonly referred to as the tax bargain. A distinctly slow and minimal improvement of human well-being in a country, is a reflection of poor governance [49]. High salaries in international institutions can fuel a similar perception.

Thirdly, countries constitutionally guarantee rights or powers to levy tax [48]. The ‘no commensurate benefits’ was softened and ‘burden’ alleviated by social welfare provisions that improve living standards. This fiscal re-distribution policy has not been undertaken, adopted or achieved in many African countries thus the burden is felt strongly. Constituent documents of international institutions are not clear: article 19 of the UN Charter states failure to pay dues results in sanctions after 2 years, although what kind of sanction is implemented remains unclear [36]. There are no limits on measures used except for reference to international standards.

Fourthly, this also affects the development of global/regional health funds and international taxes that target health concerns with a few adjustments. In order for these funds and taxes to be created and continue to exist their responsibility must rest with transparent, accountable and responsible institutions that work effectively and efficiently and make decisions that are fair and just on behalf of those that place money in their control: member states and people of the world. Since money for a health fund would be automatically earmarked for health: the only legitimate spending from this fund can be to improve global health. International fiscal legitimacy in health would allow the collection of resources from willing and compliant states and citizens satisfied with improved health outcomes.

In order to achieve health regionally and globally, it must be clear that rights require resources and health is a shared global responsibility and requires international co-operation and assistance which can only be achieved through a fiscally legitimate process. Simply reaching this point is not enough: how this legitimacy should be achieved and what the principles guiding fiscal legitimacy look like will be discussed next. Within fiscal laws and policies there are settled principles and canons that can provide a guide in collection and spending.

### Applying Principles and Canons of Taxation to Health Financing

Almost every country in the world prepares its budget using principles and canons of taxation to achieve fiscal legitimacy. This is reflected in how the state decides: what tax to collect; from whom to collect; in what amount; to whom to grant exemptions; and whether it is fair or economically feasible to collect. The state budget is prepared by economists in ministries of finance who compromise one issue for another usually to collect the most from the wealthy using the overall guiding principle of progressive tax systems before being presented in parliament. There are usually political compromises depending on the strength of the lobbyists, as a result, it will always be sub-optimal [23]. Despite the negotiations, this compromise-based budget hits certain benchmarks of tax principles, as choices must be justified at the legal, economic, social and political levels. In this real world of fiscal decision-making principles allow for the most rational compromise possible based on guiding principles.

The principles/canons in this section discuss the three main economists that developed them: Khaldun, [30, 31] Smith [24] and Ricardo [30, 31]. This sub-section will set them out to assess how international fiscal health legitimacy could be achieved when reflecting on: existing resources, new forms of tax, global taxes as well as the global and regional health institutions and funds that are being considered for health.

The first canon: justice. No one likes to pay taxes and based on this assumption, Ibn Khaldun argued for low tax rates in order that the incentive to work is not killed and taxes are paid willingly [24]. At the regional and global level this would include small amounts of membership dues to international organisations. For example, global taxes like the carbon tax should have low rates of tax of a small fixed amount. This principle of justice was later supported by David Ricardo and reiterated by Stiglitz [33] and has since been referred to as the canon of justice. It is an overall principle that ideally should guide all fiscal decision-making.

The second canon: ability to pay. Ibn Khaldun, in discussing taxpayers’ ability to pay, argued that when government is honest and people-friendly, “taxation yields a large revenue from small assessment.” [23] Smith [30, 31] supported it. It could be separated into the third canon: economy, which recommends that the cost of collecting tax should be the minimum possible for both the government and the taxpayer. This links the physical limits of both the taxpayer to the tax administrative costs of the collector. When referring to examples of new funds like the health impact fund or even the Ebola emergency fund it is important that these funds do not cost more to administer than they receive.

The fourth canon: equality or equity. Smith [30, 31] called it economic justice. At the WHO level it implies that all member countries should be allowed to vote on the projects for financing and that the process cannot and should not be driven by the decisions of the large contributors. This canon would also support the ability to pay argument that more wealthy states should pay more money into communal funds while spending gives states an equal voice. However this limited justice to only issues of economy and equality and not other areas of justice as set out by ibn Khaldun which include social justice as well.

The fifth canon: certainty. Smith argued that taxpayers should not be subjects to the arbitrariness and discretion of the tax officials, which could breed corruption [30, 31]. In the context of WHO financing it would mean that assisting low income countries in meeting their financial needs precludes arbitrary conditionalities. Global and regional health financing would require that membership dues are known in advance, fixed, clear and stable. Domestically revisions are made bianually but global processes take longer to negotiate and inserting formulas in clauses allowing for upward movement by a small percentage after a fixed period or a downward movement based on the growth of the economy may be more reliable.

The sixth canon: convenience [23] The mode and timing of tax payment should be convenient to the taxpayers. In this case the taxpayer would be member states and the collector would be the fund being set up or health institution being mandated to carry out certain work. The system in place would again reflect collections reflective of state circumstances.

The seventh canon: productivity [30, 31] The tax system should be able to gain enough revenue to avoid deficit financing. Most international institutions and states today work under deficit financing and the use of deficits has resulted in the differences in the debt burden of children.

The eighth canon: flexibility, requires that funds be re-directed when necessary. In case of health emergencies this should be within certain parameters with minimum delay. The ninth canon: simplicity requires that a system should not be complicated, be easy to understand, administer and interpret. To this Stiglitz adds the system should respond easily to changes in economic conditions [33].

The tenth canon: diversity argued by Stiglitz requires that there should be diverse sources of state income such as government engagement in business and tax. At regional and international level this would be reflective of the current health structure where revenues include memberships that are traditionally sources from the nations (most likely from tax) as well as private donations, which could be in cash or kind without undermining those allowed to make spending decisions [33].

Stiglitz also adds economic efficiency: the tax should allow for efficient allocation of resources. In the context of health financing in Africa for example, efficiency could mean allocating 15 % of the state budget to health. Coupled with transparency this could result in a tax burden easily ascertainable and politically tailored to what society considers desirable [33].

Today these principles are balanced off with each other and compromised in the fiscal and political processes leading up to tax decisions at domestic level [5, 28, 46]. In conclusion, this section discussed the absence of resource allocation and fiscal principles in allocations of resources to health globally. It looked into how one could begin to conceptualize international co-operation and development from both a health and a fiscal perspective to obtain and allocate resources to global health. It has then set out the domestic principle of fiscal legitimacy and its constituent tax principles to provide guidance on how one would collect and allocate resources. The next section will discuss health financing policy and practice in place and/or being discussed at the global and African level through the lens of the principles of taxation.

## The Current Global and African Practices and Policies of Financing Health

During the Social Development Goals (SDG) discussions, countries and regions prepared joint position papers on health and other SDG issues for discussion on the way forward past the 2015 MDG deadline. Africa was the only continent to release not only a common African position paper but also regional joint position papers setting out policies for the SDG process to consider, making them a unique continent to assess for the purposes of health financing. This section will analyse these papers in reference to resources; the obligation of international co-operation and assistance as well as fiscal legitimacy and the cannons and principles of taxation discussed in the previous section. This section will set out the international and national health financing practices currently in place with specific reference to African states.

### Domestic Health Financing Practices

National health care systems are based on either one or several of financing programs, which often co-exist simultaneously [12]. There are currently no African regional health financing systems in place and the global system is limited to international institutions such as the WHO.

National level methods used include: general revenue financing, social insurance, private insurance and out of pocket payments. Firstly, general revenue financing occurs when current country revenues are used to finance current expenditure. Either all or a portion of these state resources can then be directed to the country’s health needs through the national budget process. In most countries in the world there is a budget line for the Ministry of Health and also for Ministries of Gender and Youth that have line votes for health expenditure: both recurrent (e.g. maintenance of current health infrastructure, purchase of medication and the wage bill) as well as for development (e.g. for building new clinics and hospitals). This would be part of a system based on the principles and canons of progressivity, justice, and ability to pay [5, 28, 46].

Secondly, social insurance financing refers to a situation where groups, usually workers in formal employment, make contributions to a health care financing program such as in Kenya, where every employee and employer must make payments to the National Hospital Insurance Fund (NHIF), however this excludes those in the informal sector or the self employed as well as those below the first tax bracket. This tends to be regressive in nature as people who pay taxes (whether direct or indirect) now also pay for health however since the amount is small, it remains slightly progressive within its structure. However, where families hiring domestic help need to make payments this becomes administratively inefficient and as a result economically inefficient costing more to administer than the amount collected.

Thirdly, private insurance financing, where people choose how much to pay as a share of their income. The first form is optional and could be set up by any company or be an insurance product. Mainly middle class and wealthy individuals subscribe to them. The second form is done through corporate social responsibility of a company for its staff providing whatever services the company decides is most suitable for them and is also used by companies to reduce their tax burden. Whereas this means there may now be a clinic in an area where there was none, non-employees are still unable to access the service. The two negative impacts are that: one, the services cannot be monitored by the state and remain the choice of the company to give or take away at will, and two: the state losses tax revenue it could have allocated for health to improve the well-being of the entire population instead of the preference being shown for some citizens over and above others even within the same region.

Finally, out of pocket payments are made directly at the point of service. This is a case where a person who is not insured visits a clinic or a private hospital for a medical attention and pays in cash or by cheque, as they have no insurance. In low-income countries, more than half of health spending is from out-of-pocket payments by consumers of care. This is a very inequitable type of financing since it is harshest on those who are poor and bars individuals from accessing the kind of financial protection, which public and private insurance mechanisms afford [27]. The result of this is a highly regressive system where the principles of progressivity and justice as well as equity are completely overturned and this scenario needs to be avoided as much as possible.

### International Health Financing Practices

At the global level there are three structures currently in use as set out by Gostin: health aid, funding health strategies and international mechanisms. The first structure, health aid. This concept divides the world into donor countries and countries in need. International co-operation and assistance on which the aid hinges, is a collaboration among countries responding to health risks together and building capacity collaboratively, through cooperation, ensuring the supply of essential vaccines and medicines, or demanding fair distribution of scarce lifesaving technologies [14, 35]. The recent Ebola emergency, illustrates the use of health aid, the disease may have begun in West Africa but there were cases in the United States (USA) while east Africa remained untouched. Using the term health aid here would mean aid to both African states as well as the USA however preventing the transmission was collaborative and of benefit to all states concerned and not benevolent [14, 35]. Global governance for health must be seen as a partnership, with financial and technical assistance understood as an integral component of the common goal of improving global health and reducing health inequalities. The African Union observed, that the world has moved to a new era of shared responsibility and global solidarity [1]. The principles of equity and ability to pay ensure that those who can cater better do so and those that have a greater need receive it.

Gostin states that while all the key players internationally, the IMF, WB, UN and WHO seem to be agreeing to reforms and their harmonization however the sole measure is finance. He argues it should not be limited to finance and incremental changes, ‘but deep and lasting improvement in health and longevity” [14, 35]. As a result, the financing principles discussed above become relevant in developing a framework for financing health at both the local and global levels from both the perspective of collection as well as spending on health.

The second structure, funding health strategies internationally. There are several strategies in place; they include the prioritization of health, international co-operation through budget support and financing research that is not solely market driven. One strategy, the MDGs and now the draft of the SDGs prioritize health for low-income countries emphasizing a cross-sectoral approach. Scholars argue that the most efficient way to do this is through social insurance financing. Proponents of this system advocate achieving of universal health coverage for all people so as to mitigate the financial consequences of health catastrophes [12]. This school of thought found its way into the World Health Assembly in 2005, where a resolution was passed urging member states to work towards universal coverage and ensure that populations have access to needed health interventions without the risk of financial catastrophe. Enhancing pre-payment for health services, determining household contributions according to ability to pay and introducing risk pooling became the core guiding principles of such efforts [8]. However, the financial resources available to developing countries, which account for 90 % of the global disease burden, limit these countries from realising these efforts. Instead, donors often come into fill these voids, and key among international donors in the field of health is the World Health Organization through the principle of international co-operation and assistance.

Another strategy Gostin summarizes is the position of those financing research in diseases by stating that manufacturers do not invest in research where the markets are unpredictable to which one could add poor and therefore with a limited profitability. As a result malaria, which is the number one disease that kills people in Africa, remains relatively under-researched as compared to other diseases like HIV/AIDS. Gostin however adds that Global Alliance for Vaccines and Immunization (GAVI) is trying to reform the vaccine marketplace through Advance Market Commitment (AMC), which is a form of innovative financing designed to correct inherent weaknesses in the market [8]. However it remains unclear what principles guide the AMC [16]. Principles of international co-operation of not just states but all stakeholders (which would include individuals and institutions within a state) coupled with the canons of justice and progressivity would require that more money be spent on researching more urgent issues not more profitable ones.

A final strategy is instead of directly financing specific health projects, as it is wont to, the WHO works with governments to support their budgets for mutually agreed programs [15]. To improve financial protection in these countries instead of risk pooling health should be achieved through the ministries of health and financed by the general budget. Further, risk pooling could be achieved through the initiation of social health insurance, voluntary health insurance or community-based health insurance [15]. Widespread health inequalities still exist in areas such as infant and child mortality, maternal mortality and stunting, and in accessing health services [51]. There are wide inequities, within and between countries, in health services coverage, safe water supply and sanitation, and health outcomes [53]. Using budget support is key to building the fiscal legitimacy of a state and as a result encouraging more compliant taxpayers who see the benefits of supporting the state. This would also lead to larger payments of membership dues to international institutions with the growth of domestic revenue collection.

Regionally, African states in 2012 through international cooperation, adopted an AU resolution advocating the need for a multi-sectoral approach involving individuals, families and communities, in successful health promotion strategies [29]. It states that health financing can be progressively achieved by formulating well-planned health promotion strategies through a multi-sectoral approach involving different actors. This decision was made after following the assessment of ten African countries’ [4] National Health Promotion Strategic Action Plans and another ten countries’ [7] performance in the health sector after technical support in developing and implementing policies revealed several gaps including issue sustainable financing [34].

The third structure includes discussions on innovations like separate international health financing mechanisms; include suggestions of new taxes and funds. However they do not canvass the loss of uncollected revenues through tax evasion and aggressive tax avoidance; the use of current funds like carbon tax which is collected on global emissions but deposited in one state to be used at will or tobacco tax collected domestically but not earmarked to health-related spending. The emphasis is on new ideas instead of fixing the existing ones. These new ideas are not widespread probably firstly, due to the novel nature of the idea as well as the fact, secondly, that they have not as yet been fully developed and finally possibly due to challenges involved in adding a new mechanism to the already burdened international system.

Pogge and Hollis have proposed the Health Impact Fund referred to above [17]. This fund will use a market-based scheme to develop and distribute medications. They argue that states and stakeholders would contribute to the fund, with funding distributed to biotechnology and pharmaceutical companies that fulfill unmet need, as measured by a standardised unit such as the disability adjusted life year (DALY) [14, 35]. This approach would allow research to continue within multinational companies to make profit while simultaneously allowing the system of patenting of drugs to continue a contentious issue in Africa [18]. This fund is not in any way an emergency fund or a common pool for distribution to states for their use. This goes against both the canon of justice and ability to pay.

There have also been calls to have transaction taxes, the tobacco tax and part of the climate fund earmarked to cater for health needs. These remain interesting ideas not fleshed out in terms of the guiding principles and overall structure and it remains unclear what canons or principles will be prioritised. While some seem to propose new methods and instruments as well as institutions it is very clear that there has been a lack of proper monitoring of taxpayers, both individual as well as institutional ones, that has resulted in low tax collections and while new methods may be useful it more likely add on more complication to an already complex system.

## Recommendations

A reflection on the principles and canons in both the right to health as well as finance coupled with the discussion on principles and practice globally and in Africa shows that although there is no clear discussion on the principles, references are constantly being made to them directly or indirectly in circles discussing health financing. It is therefore recommended that:

Firstly, there must be recognition both within human rights and finance that ‘rights require resources’. This includes and is not limited to: conventions, resolutions as well as international fiscal contracts and contracts with international financial institutions.

Secondly, there is a need for further research into international co-operation and assistance. The language of ‘health aid’ should be amended to recognise international co-operation and assistance and could instead be re-phrased as ‘mutual health assistance’.

Thirdly, fiscal legitimacy should be recognized at regional and global levels to build in the principles of good governance as well as efficiency, effectiveness, fairness and justice at all levels.

Fourthly, reference should be made to the principles using agreed definitions of what they involve for purposes of consistency and measuring progressive financing of health. Terminology already discussed could provide a starting point.

Fifthly, more reflection needs to take place on the existing issues of international tax evasion and avoidance as a source of untapped income before making a foray into new global taxes and mechanisms. However a regional or sub-regional emergency or common funds as suggested by the North African states could be funded by existing international taxes.

Finally, financing health must go through the state and must take national priorities in account in order that fiscal legitimacy is built with the state to encourage the continued cycle of tax payments to fund health. Regionally and globally, state priorities must take precedence to build fiscal legitimacy in the global or regional mechanisms being set up and ensure continued state support.

## Conclusion

The process of taking stock allows one to see where we are and hopefully the next step. This paper has picked up the point where health financing globally sits through the work of Gostin and explored the next steps that could be taken in order to realise the right to health. It has drawn together principles and practice from an African perspective while referring to the global positions to create a picture that demonstrates that in financing health globally and regionally, one must firstly, address issues of global and regional principles and structures that already exist before adding in more complex mechanisms and secondly, to use existing principles to rectify and build into the harmonisation processes being undertaken to achieve health as a global concern.
